# A comparative assessment of the glucose monitor (SD Codefree) and auto analyzer (BT-3000) in measuring blood glucose concentration among diabetic patients

**DOI:** 10.1186/s13104-017-2789-0

**Published:** 2017-09-06

**Authors:** Osman Nabayire Kanwugu, Gideon Kofi Helegbe, Paul Armah Aryee, Nathaniel Amigamsa Akontatiba, Jacob Ankrah, Nsoh Godwin Anabire, Frank Anaba, Benjamin Ahenkora

**Affiliations:** 1 Department of Biochemistry & Molecular Medicine, School of Medicine and Health Sciences, University for Development Studies, P. O. Box TL 1883, Tamale, Ghana; 2 Department of Community Nutrition, School of Allied Health Sciences, University for Development Studies, P. O. Box TL 1883, Tamale, Ghana; 3 Department of Applied Chemistry & Biochemistry, University for Development Studies, P. O. Box 24, Navrongo, Ghana; 4 Department of Hematology, Tamale Central Hospital-Tamale, Tamale, Ghana; 5 Bolgatanga Regional Hospital, Upper East, Bolgatanga, Ghana

**Keywords:** Diabetes mellitus, Hyperglycaemia, Glucometer, Auto analyzer

## Abstract

**Objective:**

The aim of this study was to determine how well the measurements from a glucometer (SD Codefree) correlated with those from a standard auto analyser (BT-3000) using blood samples from diabetic and non-diabetic patients at the Bolgatanga Regional Hospital in Ghana. A cross-sectional study was conducted with a total of 150 randomly selected patients; 100 diabetic patients (4 type 1 and 96 type II) and 50 non diabetic patients. Ante-cubital venous and finger pricked blood samples were obtained from the patients following standard procedures, and blood glucose concentrations were determined using the two methods respectively.

**Results:**

Data generated was entered and analysed using SPSS version 20. The mean glucose concentration for the diabetic patients (n = 100) using the glucometer were not significantly different from that of the auto analyser (10.16 ± 3.708 mmol/L vs. 9.458 ± 3.204 mmol/L, p = 0.154), though the glucometer generally overestimated the glucose concentration. Similarly, readings for non-diabetics were comparable between the two methods (5.286 ± 0.477 mmol/L vs. 5.092 ± 0.525 mmol/L, p = 0.057). The correlation between the two methods was good and highly significant (r = 0.862, p < 0.001) with both methods depicting high sensitivity and specificity in measuring blood glucose levels among diabetics as indicated by the ROC curve.

**Electronic supplementary material:**

The online version of this article (doi:10.1186/s13104-017-2789-0) contains supplementary material, which is available to authorized users.

## Introduction

Diabetes mellitus is a group of metabolic diseases characterized by elevated blood glucose concentration (hyperglycaemia) resulting from either defects in insulin secretion by the pancreas, insulin action or both [1]. While type-I DM occurs mostly in individuals less than 18 years of age and accounts for only 5–10% diabetics, type-II DM has a common occurrence in people over 40 years of age and accounts for 90–95% of individuals with diabetes [1–3]. Diabetes mellitus is a cause of morbidity, disability and mortality worldwide. Diabetic-hyperglycaemia is often marked by polyuria, polydipsia, weight loss and sometimes polyphagia and blurred vision [4]. It has been estimated that the total number of diabetics worldwide will rise from 171 million as at 2000 to 366 million in 2030 with more than 85% of them living in low and middle income countries [5].

Increasingly, glycaemic control is being recognized as a priority in the treatment of critically ill diabetic patients as it has helped to significantly reduce mortality and morbidity [6, 7]. The acquisition of information about blood glucose concentration is an important parameter for the establishment of much diagnostic as well as vital therapy [2]. Glucometers are devices developed to measure glycemia of capillary blood obtained through finger or heel puncture using a lancet or hypodermic needle. They are automatic, fast and easy to use and determine the blood glucose concentration mostly by means of either photometric or electrochemical reactions [8]. Currently, many diabetics achieve SMBG in two general ways of measuring their blood glucose; the glucometer and the laboratory-based chemistry auto analyser. Although the latter method is perceived as more reliable and accurate, the use of the glucometer is rather preferred because it is portable and convenient to use. However, for effective SMBG it is imperative to have reliable and accurate measure of blood glucose level. In the recent years, conflicting results have been reported with regard to the accuracy of these devices [15]. Currently in Ghana, however, little to no literature is available on the accuracy of these devices. Meanwhile, SD codefree glucometer is one of the main glucometers used in Ghana including the Bolgatanga Regional Hospital. This calls to question the need to ascertain the accuracy and reliability of the glucometer in comparison with the standard laboratory method. This current study is an attempt to explore the accuracy of one of the many brands of glucometers that have flooded the Ghanaian market.

## Main text

### Methods

#### Study design

The study design was cross-sectional comprising of a total of 150 randomly selected patients; 100 diabetics (4 type 1 and 96 type II) and 50 non diabetic. The sample size was determined with Cochran’s sample size determination formula using diabetes prevalence of 6.5% and 95% confidence interval.

#### Study area

The study was carried out in Bolgatanga, the capital town of the Upper East Region of Ghana and co-terminus with the Bolgatanga Municipal Assembly. It is situated at the centre of the region and to the north-eastern part of Ghana. It has a total land area of 729 sq km [9]. The Municipal has a total of 1698 reported cases of DM [10].

#### Subject recruitment

Diabetic patients (as stated in their folders) attending the diabetic clinic of the Bolgatanga Regional Hospital were randomly selected for the study. In addition, non-diabetic patients (per their medical records) attending the outpatients’ department (OPD) and eye clinic of the hospital were also randomly recruited as controls. At each department visited, the study and its significance were explained to all patients and medical records of all who volunteered to be part were reviewed, those with other known metabolic disorders were excluded from the study.

#### Data/sample collection

After consent was obtained from participants, socio-demographic information including sex, age and type of DM, were collected by the authors with the aid of questionnaires during the data collection process. Those involved in the questionnaire administration were given a 1-day training, and questionnaire was pre-tested. Blood samples were collected by a trained medical laboratory scientist from the ante cubital vein and capillary of fingers for the reference glucose oxidase method and glucometer measurements respectively, after an overnight fast (8–14 h), and following standard procedures as described by [11].

#### Measurement of glucose level using the glucometer

Glucose level in capillary blood was measured with the glucometer (SD Codefree) using standard procedures described by the manufacturer.

#### Measurement of glucose level using the auto analyser

Glucose level in venous blood was also measured with the auto analyser (BT-3000) following standard procedures described by the manufacturer.

#### Data analysis

The data collected from the study was analysed using SPSS version 20 and results presented as mean ± standard deviation. The comparison of the mean values from the two methods was done using independent *t* test at a 95% confidence interval and the differences were considered statistically significant if p < 0.05 (Additional file 1).

### Results

The blood glucose level of both diabetic and non-diabetic patients enrolled in this study was determined simultaneously with the glucometer (SD-Codefree) and auto analyser (BT-3000) in the laboratory of the Bolgatanga regional hospital. Results obtained did not show any significant statistical difference between blood glucose readings using both methods in diabetics and non-diabetics alike. However, it was notable that the glucometer tended to slightly over-estimate the measurements in all cases, averagely by 0.721 mmol/L in diabetics and 0.390 mmol/L in non-diabetics.

Among the diabetics (Table 1), the results showed no statistically significant difference (p = 0.142) between glucose levels obtained with the glucometer (10.135 ± 3.708 mmol/L) and the auto analyser (9.411 ± 3.224 mmol/L). When glucose levels in this category was assessed according to age, the results did not show any statistically significant difference in the measurements between the glucometer and the auto analyser within patients aged 20–40 (p = 0.680), 41–60 (p = 0.239) and 61–80 (p = 0.452). Also, the difference in glucose levels obtained using the two methods among type I and type II diabetics were highly comparable (p values of 0.621 and 0.158 respectively) even though measurements were slightly higher among type II diabetics.

**Table 1:** Mean concentration of glucose of diabetic patients

	Mean glucose concentration (mmol/L) ± SD		P value
	Glucometer (n = 100)	Auto analyzer (n = 100)	
Mean FBS	10.135 ± 3.708	9.411 ± 3.224	0.142
Age (years)			
20–40	9.827 ± 3.322	9.288 ± 2.683	0.680
41–60	10.232 ± 3.839	9.434 ± 3.264	0.239
61–80	10.073 ± 3.703	9.441 ± 3.404	0.452
Sex			
Female	9.979 ± 3.451	9.310 ± 3.396	0.455
Male	10.604 ± 4.437	9.711 ± 2.973	0.206
Type of diabetes			
Type I	8.525 ± 2.886	7.585 ± 2.171	0.621
Type II	10.202 ± 3.736	9.487 ± 3.246	0.158

Source: Field Survey Level of significance, p < 0.05

In the non-diabetic group (Table 2), mean glucose level of 5.338 ± 0.538 mmol/L was obtained with the glucometer while the auto analyser yielded a mean glucose level of 4.948 ± 0.726 mmol/L. Comparing the two means with t-test yielded a p-value of 0.003. In this same group, when blood glucose level was assessed according to age of patients, a significant difference was observed between the glucose level of patients aged 20-40 using the glucometer and the auto analyser (5.450 ± 0.544 mmol/L cf. 4.893 ± 0.765 mmol/L, p = 0.004). However, among patients aged 41–60, the results were not significantly different between measurements with the glucometer and that of the auto analyser (5.265 ± 0.552 mmol/L cf. 4.978 ± 0.674 mmol/L, p = 0.150), likewise that observed among patients aged 61-80 (4.975 ± 0.150 mmol/L cf. 5.155 ± 0.877 mmol/L, p = 0.711). Similarly, results according to gender were not significantly different between measurements by the glucometer and the auto analyser among the female patients (p = 0.050) as well as among the males (p = 0.162).

**Table 2:** Mean glucose concentrations of non-diabetic patients (n = 50)

	Mean glucose concentration (mmol/L) ± SD		P value
	Glucometer (n = 50)	Auto analyzer (n = 50)	
FBS	5.338 ± 0.538	4.948 ± 0.726	0.003*
Age (years)			
20–40	5.450 ± 0.544	4.893 ± 0.765	0.004*
41–60	5.265 ± 0.552	4.978 ± 0.674	0.150
61–80	4.975 ± 0.150	5.155 ± 0.877	0.711
Sex			
Female	5.503 ± 0.458	5.059 ± 0.714	0.162
Male	5.068 ± 0.562	4.767 ± 0.727	0.050

Source: Field Survey * Statistically significant Level of significance, p < 0.05

**Fig. 1:**
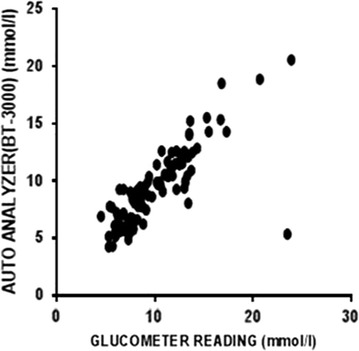
Correlation between glucose levels measured using glucometer and BT-3000 auto analyser

All the results indicated a good and significant correlation between the glucometer and the auto analyser (Pearson correlation coefficient, r = 0.862, p < 0.001) as illustrated in Fig. 1. When the results were presented on a ROC curve, the area under the curve with regards to the glucometer was 0.962 while that of the auto analyser was 0.950 (Additional file 2).

### Discussion

Self-monitoring of blood glucose (SMBG) using glucometers as an essential part of diabetes care has become prominent probably because it is a very practical and cost effective approach to diabetic self-care [2]. In recent years, conflicting results have been reported with regard to the accuracy of these devices [12–14]. The aim of this study was therefore to assess the efficiency of the glucometer by comparing it with the standard glucose oxidase/peroxidase colorimetric method used in measuring blood glucose concentration in the laboratory.

Much as the results between the two methods were not statistically significant irrespective of the diabetic status of patients, the glucometer tended to over-estimate the measurements in almost all the cases. These results corroborate observations made in other studies [15–17]. However, other studies conducted by [18] and [19] reported that the glucometer generally produced lower glucose readings though there was a good correlation between the glucometer and the auto analyser. The results above generally show that capillary blood glucose may not be reproducible as venous blood glucose concentration. Although the glucometers as well as the standards for comparisons in these studies are different in terms of brands, the underlining working principles [19] are the same and therefore make the results of this current study comparable to findings of these studies. In the body, blood glucose levels in the capillary differ from that in the veins. Venous plasma glucose level is the estimated glucose after utilization of glucose by tissues and depends on the extent of tissue extraction of glucose as well as effect of insulin, glucagon, growth hormone and cortisone [16]. The difference in blood glucose levels recorded by the glucometer and the auto analyser maybe accounted for partly by these factors mentioned above as well as changes in temperature and humidity and altitude [14]. The results from this study showed no influence of age, gender and type of DM on the glucose readings recorded by both methods.

The results show a near perfect correlation between glucose levels generated by the glucometer and the auto analyser. Thus, the glucometer used in this study is relatively accurate at measuring the glucose level of the patients and irrespective of their diabetes status. These findings correspond to those from studies conducted by [8] and [20] who found similar levels of correlation between the glucometer and auto analyser. However, in a similar study conducted in Uganda by [19] to determine the accuracy of three glucometer systems, only one (the system used by One Touch™, LifeScan Inc. and Milpitis) out of the three showed a good correlation with a secondary standard, pre-standardized by the laboratory based auto analyser. In contrast, a study conducted by [21] found a weak correlation (r = 0.275) between a glucometer and the laboratory based method in determining neonatal hypoglycaemia. In light of this, it would be wrong to assume that any brand of glucometer is accurate in its measurements until it has been standardised. Clearly, standardisation of glucometer brands against trusted methods is imperative to forestall obtaining results that may be spurious and could jeopardise the health of the patient.

A rather interesting finding observed in this current study is that the ROC curve showed the glucometer to be an even better method as indicated by the area under the curve than the auto analyser. The area under the ROC curve represents a measure of discrimination, thus, the ability of the test to classify correctly those with and without the disease. The closer the area under the ROC curve is to 1, the more accurate the test [23]. Although over the years there has been advances in technology that has improved the glucometer, the variation in measurements that shows the glucometer to be more accurate in this study is most likely to be as a result of pre-analytical errors that may have occurred in the usage of the auto analyser. Nevertheless, despite the apparent accurate results observed this current study cannot be a basis to generalize for all glucometers on the market.

Overall, though the results of this study indicated that the glucometer can still be used for SMBG as part of diabetes management and care, it is, however, crucial that standardization of glucometer be given attention in policies geared toward management and control of diabetes since clearly, the accuracy of SMBG depends to a large extent on the instrument used as well as the user [22].

### Conclusion

We conclude that the glucometer (SD Codefree) is as accurate as the auto analyser and therefore can be conveniently used as a rapid easy-to-use alternative. However, it is recommended that further studies be carried out using multiple glucometer brands.

## Limitation

In this study, only the SD Codefree brand of glucometer was used and therefore the results cannot be generalized for all brands of glucometers found on the Ghanaian market.

## Additional files

**Additional file 1.** Dataset: Data used for the analysis whose results are presented in this manuscript are carried out.

**Additional file 2.** ROC Curve: A graph presenting a ROC curve for both the glucometer and auto-analyzer.

## Abbreviations

Cf: compared to
DM: diabetes mellitus
ROC: receiver operating characteristic
SMBG: self-monitoring of blood glucose
SPSS: Statistical Package for Social Sciences
Vs: versus
