# Supplementary figures and images for: A comparative assessment of the glucose monitor (SD Codefree) and auto analyzer (BT-3000) in measuring blood glucose concentration among diabetic patients
Source: BMC Res Notes. 2017 Sep 6;10:453. doi: 10.1186/s13104-017-2789-0 (PMC5588599; doi:10.1186/s13104-017-2789-0)

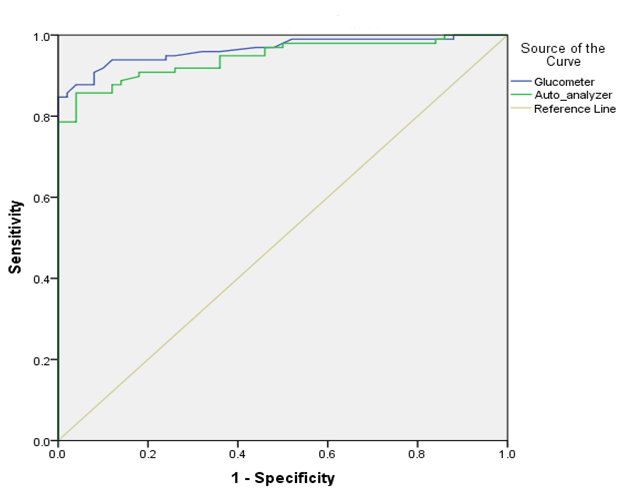

Supplement: Supplementary file 2 — Additional file 2. ROC Curve: A graph presenting a ROC curve for both the glucometer and auto-analyzer. [file 13104_2017_2789_MOESM2_ESM.png]
